# Sexual abuse, abortion and public health in Brazil: when moral judgment accentuates inequities

**DOI:** 10.1590/0034-7167.202477suppl0401

**Published:** 2024-08-19

**Authors:** Ricardo de Mattos Russo Rafael, Livia Angeli-Silva, Ivone Evangelista Cabral, Roberta Georgia Sousa dos Santos

**Affiliations:** I Universidade do Estado do Rio de Janeiro. Rio de Janeiro, Rio de Janeiro, Brazil; II Universidade Federal da Bahia. Salvador, Bahia, Brazil; III Universidade Federal do Rio de Janeiro. Rio de Janeiro, Rio de Janeiro, Brazil

Here is an academic-political editorial that aims to call on the scientific field to reflect on the dangers of decisions regarding the right to abortion under moral rules, since there is no evidence that such a perspective translates into practices of care, protection of childhood and gender equity - elements that are so urgent and necessary for nursing. Being even more concrete, this editorial addresses - and, in advance, repudiates - Bill 1,904/2024, presented to the Federal Chamber of Deputies, with the aim of modifying the Brazilian Penal Code.

These changes equate abortion during pregnancy over 22 weeks to homicide, regardless of the circumstances, while also criminalizing abortion by healthcare professionals. The approval of this Bill would annul legislation that provides for the right to abortion in cases where pregnant women’s life is at risk, cases of fetuses diagnosed with anencephaly and, as the central point of this editorial, in cases of rape for a significant part of the population. Furthermore, by equating abortion to the crime of homicide, a rape victim who underwent abortion procedures could be sentenced to a prison sentence of up to 20 years, whereas rape perpetrators could be sentenced to a prison sentence of six to ten years.

It turns out that legal abortion in pregnancies longer than 22 weeks is a resource used by people who face barriers to accessing the rights guarantee system (including the health system). Essentially, children under 14 use this resource, since rape in childhood is a phenomenon, in general, resulting from systematic and long-lasting practices of sexual abuse in the family environment. Furthermore, a significant portion of the population subscribes to a conservative worldview that prohibits discourse in favor of sexual health education at school and dialogue about human sexuality in childhood and adolescence. As a result, girls especially have limited knowledge about the body changes that occur since pre-puberty, and pregnancy diagnosis can be made late^(1)^.

Data from the Notifiable Diseases Information System (SINAN - *Sistema de Informações de Agravos de Notificação*) reports 470,947 records of violence against female children and children under 14 years of age in Brazil between 2013 and 2022. Of these records, 138,636 (29.43%) were for rapes, of which alleged perpetrators were the father (11.99%; n=16,625), the stepfather (13.24%; n=18,355), the brother (2.78%; n=3,853), other caregivers (0.80%; n=1,116) and friends and acquaintances (25.50%; n=35,361). Only 13,196 (9.52%) of rape records were committed by strangers in this age group. Under the terms of the Child and Adolescent Statute, the family, the community, society in general and the public authorities must ensure, with absolute priority, the realization of the rights to a life with health, dignity and respect.

It appears that the family and home, places idealized as protected and safe, have, in fact, served as a privileged place for perpetration of abuses and their systematic concealment. Therefore, it is not difficult to imagine that some pregnancies are only publicly revealed at 22 weeks or more. Even so, sometimes the moralizing discourse of “child protection” used to condemn abortion does not gain the same proportion in cases of child victims of rape, since, even with consent, all sexual relations with minors under 14 are considered rape of a vulnerable person in Brazil. There were 219,055 births of children under 14 years old registered in the Ministry of Health’s Live Birth System between 2013 and 2022.

This scenario demonstrates that the rape of vulnerable people is a reality and has been treated in a moralizing and neglected manner, and the Bill that gave rise to this editorial, by disregarding the entire complexity of the phenomenon, contributes to intensifying inequities. Good and evil, so used in the debate on violence, are not ontological phenomena, they are political and, consequently, outlined in everyday life in society^(2)^. Concerning girls’ and women’s lives, this violence intersects with other markers, such as race/color. Black and indigenous women are more likely (three times and 16.84 times, respectively) to be hospitalized for causes related to pregnancy, childbirth and the postpartum period when compared to white women, according to hospitalization rates calculated from the Hospital Information System and the 2022 Demographic Census.

Therefore, the intensification of punitive provisions for the practice of abortion will not make this phenomenon cease to exist. Even though obedience to legal and social rules has meaning for society, it does not necessarily have a direct correspondence with the way people behave in a real and particular situation^(3)^. Therefore, the usefulness of asking whether we are “against or in favor of abortion” or the curtailment of these rights is small - if it really exists at all. In the concrete reality where life operates, but in different ways in terms of opportunities, abortion is part of the daily lives of Brazilians from different ideological backgrounds, as demonstrated by the Brazilian National Abortion Survey^(4)^. Operating it as a human right is an urgent need for producing more equitable, universal care policies decided by women.
