# Supplementary material for: Sexual abuse, abortion and public health in Brazil: when moral judgment accentuates inequities
Source: Rev Bras Enferm. 2024 Aug 19;77(Suppl 4):e77suppl0401. doi: 10.1590/0034-7167.202477suppl0401 (PMC11338527; doi:10.1590/0034-7167.202477suppl0401)
Supplement: Translation

# Translation: Abuso sexual, aborto e saúde pública no brasil: quando o julgamento moral acentua as iniquidades

**DOI:** 10.1590/0034-7167.202477suppl0401pt

**Authors:** Ricardo de Mattos Russo Rafael, Livia Angeli-Silva, Ivone Evangelista Cabral, Roberta Georgia Sousa dos Santos

**Affiliations:** I Universidade do Estado do Rio de Janeiro. Rio de Janeiro, Rio de Janeiro, Brasil; II Universidade Federal da Bahia. Salvador, Bahia, Brasil; III Universidade Federal do Rio de Janeiro. Rio de Janeiro, Rio de Janeiro, Brasil

Eis aqui um editorial acadêmico-político que tem como objetivo convocar o campo científico a refletir sobre os perigos das decisões relativas ao direito ao aborto sob réguas morais, uma vez que não há evidências de que tal perspectiva se traduza em práticas de cuidado, proteção à infância e equidade de gênero - elementos tão urgentes e necessários à enfermagem. Sendo ainda mais concretos, este editorial aborda - e, de antemão, repudia - o Projeto de Lei (PL) 1.904/2024, apresentado à Câmara dos Deputados Federais, com o intuito de modificar o Código Penal Brasileiro.

Tais mudanças equiparam o aborto na gestação com mais de 22 semanas a homicídio, independentemente das circunstâncias, ao mesmo tempo em que também criminaliza a realização de pelos profissionais de saúde. A aprovação desse PL tornaria letra morta a legislação que prevê o direito ao aborto para os casos de risco de vida para a gestante, os casos de fetos com diagnóstico de anencefalia e, como ponto central deste editorial, em casos de estupro para parte expressiva da população. Ademais, ao equiparar o aborto a crime de homicídio, uma vítima de estupro que se submeteu a procedimentos de aborto poderá ter decretada uma pena de reclusão que atinge 20 anos, enquanto que o perpetrador do estupro poderia ter pena de reclusão de seis a dez anos.

Ocorre que o aborto legal em gestações com mais de 22 semanas é recurso utilizado por pessoas que enfrentam barreiras de acesso ao sistema de garantia de direitos (inclusive, o sistema de saúde). Essencialmente, crianças menores de 14 anos utilizam este recurso, já que o estupro na infância é fenômeno, no geral, consequente de práticas sistemáticas e duradouras de abuso sexual no ambiente familiar. Ademais, uma faixa expressiva da população é signatária de uma visão de mundo conservadora que interdita o discurso em favor da educação em saúde sexual na escola e do diálogo sobre a sexualidade humana na infância e adolescência. Como resultado, especialmente as meninas possuem conhecimento limitado sobre as mudanças corporais que acontecem desde a pré-puberdade, e o diagnóstico da gravidez pode ser feito tardiamente^(1)^.

Dados do Sistema de Informações de Agravos de Notificação (SINAN) informam 470.947 registros de violências contra crianças do sexo feminino e menores de 14 anos no Brasil entre 2013 e 2022. Desses registros, 138.636 (29,43%) foram por estupros, dos quais os alegados autores foram o pai (11,99%; n=16.625), o padrasto (13,24%; n=18.355), o irmão (2,78%; n=3.853), outros cuidadores (0,80%; n=1.116) e amigos e conhecidos (25,50%; n=35.361). Apenas 13.196 (9,52%) dos registros de estupro foram cometidos por desconhecidos nessa faixa etária. Nos termos do Estatuto da Criança e do Adolescente, é dever da família, da comunidade, da sociedade em geral e do poder público assegurar, com absoluta prioridade, a efetivação dos direitos a uma vida com saúde, dignidade e respeito.

Constata-se que a família e o domicílio, lugares idealizados como protegidos e seguros, têm, na verdade, servido como lugar privilegiado para a perpetração de abusos e sua sistemática ocultação. Deste modo, não é difícil imaginar que parte das gestações somente seja publicamente revelada com 22 semanas ou mais. Ainda assim, por vezes, o discurso moralizante de “proteção à infância” usado para condenar o aborto não ganha a mesma proporção para os casos das crianças vítimas de estupro, já que, mesmo com consentimento, toda relação sexual com menores de 14 anos é considerada estupro de vulnerável no Brasil. Foram 219.055 partos de crianças menores de 14 anos registrados no Sistema de Nascidos Vivos do Ministério da Saúde entre 2013 e 2022.

Tal cenário demonstra que o estupro de vulneráveis é dado da realidade e tem sido tratado de modo moralizante e negligenciado, e o PL que deu causa a este editorial, ao desconsiderar toda a complexidade do fenômeno, contribui para intensificação de iniquidades. O bem e o mal, tão utilizados no debate sobre as violências, não são fenômenos ontológicos, eles são políticos e, consequentemente, delineados no cotidiano da vida em sociedade^(2)^. No que tange à vida das meninas e mulheres, essas violências se interseccionam com outros marcadores, como o de raça/cor. Mulheres negras e indígenas têm mais chances (três vezes e 16,84 vezes, respectivamente) de serem internadas por causas relacionadas à gravidez, ao parto e ao puerpério, quando comparadas às mulheres brancas, conforme taxas de internações calculadas a partir do Sistema de Informação Hospitalar e do Censo Demográfico de 2022.

Assim, a intensificação de dispositivos punitivos para a prática do aborto não fará esse fenômeno deixar de existir. Ainda que a obediência ao regramento jurídico e social tenha significado para a sociedade, ela não necessariamente tem correspondência direta com a maneira como as pessoas se comportam em uma situação real e particular^(3)^. Desse modo, é pequena - se é que é realmente existente - a utilidade da pergunta se somos “contra ou a favor do aborto” ou o cerceamento desses direitos. Na realidade concreta onde a vida opera, mas de modo distinto em oportunidades, o aborto faz parte do cotidiano de brasileiros de diferentes matrizes ideológicas, tal como demonstra a Pesquisa Nacional de Aborto^(4)^. Operá-lo como um direito humano é uma necessidade urgente para a produção de políticas de cuidado mais equânimes, universais e decidas por mulheres.

# Translation: Abuso sexual, aborto y salud pública en Brasil: cuando el juicio moral acentúa las inequidades

**DOI:** 10.1590/0034-7167.202477suppl0401es

**Authors:** Ricardo de Mattos Russo Rafael, Livia Angeli-Silva, Ivone Evangelista Cabral, Roberta Georgia Sousa dos Santos

**Affiliations:** I Universidade do Estado do Rio de Janeiro. Rio de Janeiro, Rio de Janeiro, Brasil; II Universidade Federal da Bahia. Salvador, Bahia, Brasil; III Universidade Federal do Rio de Janeiro. Rio de Janeiro, Rio de Janeiro, Brasil

He aquí un editorial académico-político que pretende convocar al campo científico a reflexionar sobre los peligros de decisiones sobre el derecho al aborto bajo reglas morales, ya que no hay evidencia de que tal perspectiva se traduzca en prácticas de cuidado, protección infantil y equidad de género, elementos tan urgentes y necesarios para la enfermería. Siendo aún más concreto, este editorial aborda - y, de antemano, repudia - el Proyecto de Ley (PL) 1.904/2024, presentado a la Cámara Federal de Diputados, con el objetivo de modificar el Código Penal brasileño.

Estos cambios equiparan el aborto durante el embarazo de más de 22 semanas con el homicidio, independientemente de las circunstancias, al tiempo que penalizan el aborto realizado por profesionales de la salud. La aprobación de este PL anularía la legislación que prevé el derecho al aborto en los casos en que la vida de la mujer embarazada esté en riesgo, casos de fetos diagnosticados con anencefalia y, como punto central de este editorial, en casos de violación para una parte importante de la población. Además, al equiparar el aborto con el delito de homicidio, una víctima de violación que se sometiera a procedimientos de aborto podría ser condenada a una pena de prisión de hasta 20 años, mientras que el autor de la violación podría ser condenado a una pena de prisión de seis a diez años.

Resulta que el aborto legal en embarazos mayores a 22 semanas es un recurso utilizado por personas que enfrentan barreras para acceder al sistema de garantía de derechos (incluido el sistema de salud). Básicamente, los niños menores de 14 años utilizan este recurso, ya que la violación en la infancia es un fenómeno, en general, resultante de prácticas sistemáticas y duraderas de abuso sexual en el ámbito familiar. Además, una porción significativa de la población suscribe una cosmovisión conservadora que prohíbe el discurso a favor de la educación sobre salud sexual en la escuela y el diálogo sobre la sexualidad humana en la infancia y la adolescencia. Como resultado, especialmente las niñas tienen un conocimiento limitado sobre los cambios corporales que ocurren desde la prepubertad, y el diagnóstico de embarazo puede realizarse tarde^(1)^.

Datos del Sistema de Información de Enfermedades de Declaración Obligatoria (SINAN - *Sistema de Informações de Agravos de Notificação*) reportan 470.947 registros de violencia contra niñas y niños menores de 14 años en Brasil entre 2013 y 2022. De estos registros, 138.636 (29,43%) fueron por violaciones, de las cuales los presuntos autores fueron el padre (11,99%; n=16.625), el padrastro (13,24%; n=18.355), el hermano (2,78%; n =3.853), otros cuidadores (0,80%; n=1.116) y amigos y conocidos (25,50%; n=35.361). Sólo 13.196 (9,52%) de los registros de violación fueron cometidos por desconocidos en este grupo de edad. En los términos del Estatuto del Niño y del Adolescente, es deber de la familia, la comunidad, la sociedad en general y los poderes públicos garantizar, con absoluta prioridad, la realización de los derechos a una vida con salud, dignidad y respeto.

Parece que la familia y el hogar, lugares idealizados como protegidos y seguros, han servido, en realidad, como lugar privilegiado para la perpetración de abusos y su ocultamiento sistemático. Por lo tanto, no es difícil imaginar que algunos embarazos sólo se revelan públicamente a las 22 semanas o más. Sin embargo, a veces, el discurso moralizante de “protección infantil” utilizado para condenar el aborto no cobra la misma proporción en los casos de niños víctimas de violación, ya que, incluso con consentimiento, toda relación sexual con niños menores de 14 años es considerada violación de una persona vulnerable en Brasil. Se registraron 219.055 nacimientos de niños menores de 14 años registrados en el Sistema de Nacido Vivo del Ministerio de Salud entre 2013 y 2022.

Este escenario demuestra que la violación de personas vulnerables es una realidad y ha sido tratada de manera moralizante y desatendida, y la PL que dio origen a este editorial, al desconocer toda la complejidad del fenómeno, contribuye a la intensificación de las inequidades. El bien y el mal, tan utilizados en el debate sobre la violencia, no son fenómenos ontológicos, son políticos y, en consecuencia, se perfilan en la vida cotidiana de la sociedad^(2)^. En lo que respecta a la vida de niñas y mujeres, esta violencia se cruza con otros marcadores, como la raza/color. Las mujeres negras e indígenas tienen más probabilidades (tres veces y 16,84 veces, respectivamente) de ser hospitalizadas por causas relacionadas con el embarazo, el parto y el puerperio, en comparación con las mujeres blancas, según las tasas de hospitalización calculadas a partir del Sistema de Información Hospitalaria y el Censo Demográfico de 2022.

Por tanto, la intensificación de las disposiciones punitivas por la práctica del aborto no hará que este fenómeno deje de existir. Si bien la obediencia a las normas legales y sociales tiene significado para la sociedad, no necesariamente tiene una correspondencia directa con la forma en que las personas se comportan en una situación real y particular^(3)^. Por lo tanto, la utilidad de preguntar si estamos “en contra o a favor del aborto” o de la restricción de estos derechos es pequeña, si es que realmente existe. En la realidad concreta donde opera la vida, pero de diferentes maneras en términos de oportunidades, el aborto forma parte del cotidiano de brasileños de diferentes orígenes ideológicos, como lo demuestra la Encuesta Nacional sobre el Aborto^(4)^. Operarlo como un derecho humano es una necesidad urgente para la producción de políticas de cuidado más equitativas y universales decididas por las mujeres.
